# Pandemic ethics and beyond: Creating space for virtues in the social professions

**DOI:** 10.1177/09697330231177421

**Published:** 2023-07-06

**Authors:** Sarah Banks

**Affiliations:** Department of Sociology, 3057 Durham University, Durham, UK

**Keywords:** Covid-19, virtue ethics, professional discretion, social workers

## Abstract

**Background:**

During the pandemic, social and health care professionals operated in ‘crisis conditions’. Some existing rules/protocols were not operational, many services were closed/curtailed, and new ‘blanket’ rules often seemed inappropriate or unfair. These experiences provide fertile ground for exploring the role of virtues in professional life and considering lessons for professional ethics in the future.

**Research design and aim:**

This article draws on an international qualitative survey conducted online in May 2020, which aimed to explore the ethical challenges experienced by social workers during Covid-19.

**Participants and research context:**

607 social workers responded from 54 countries, giving written online responses. This article first summarises previously published findings from the survey regarding the range of ethical challenges experienced, then develops a new analysis of social workers’ accounts of ethically challenging situations from a virtue ethics perspective. This analysis took a narrative ethics approach, treating respondents’ accounts as stories featuring the tellers as moral agents, with implicit or explicit implications for their professional ethical identity and character. The article is illustrated with accounts from the 41 UK respondents, drawing particularly on two case examples.

**Ethical considerations:**

Ethical approval was gained from Durham University and anonymity was ensured for participants.

**Findings/results:**

This article explores the nature of the ethical space created during the pandemic showing how practitioners were able to draw more on ‘inner resources’ and professional discretion than usual, displaying virtues such as professional wisdom, care, respectfulness and courage as they took account of the specific contexts of their work, rather than simply adhering to blanket rules.

**Conclusion:**

Exploring practice through a virtue ethical lens provides valuable lessons for ‘building back better’ in social and health care professions.

## Introduction

This article has a focus on the virtues of social and health care professionals. Drawing on a broadly neo-Aristotelian view of virtue ethics, I regard a virtue as a good character trait or moral quality of a person that entails a disposition to feel, think and act to promote human and ecological flourishing, involving both the motivation to act well and, typically, the achievement of good ends. Virtue ethics takes the character of the person as a moral agent as its central focus, as opposed to consequences or actions.^1,2^

## The importance of virtues during Covid-19 – some views from the literature

Much scientific and political debate about how to respond to Covid-19 featured macro-level consequentialist arguments weighing up harms and benefits of various courses of action. Nevertheless, deontological, rights-based and virtue ethical concerns were also present in public discourse. Each blanket restriction introduced by governments or institutions for the ‘public good’ engendered debates not only about weighing public health against economic damage, but also protection of vulnerable people against the freedom of others, and justice in distributing scarce resources against being caring to particular people in need. According to virtue ethics, being just and caring in particular situations, for example, would be regarded as manifestations of the virtues of justice and care.

Not surprisingly, there have been several calls in the literature to recognise the importance of virtues in general, and some virtues in particular, in coping with and responding to challenges generated by the pandemic.^3–6^ This literature highlights the value of virtue ethics in both describing and prescribing the moral qualities, attitudes and actions of politicians, service providers and citizens. This entails shifting our focus from macro-ethical concerns at population level to micro-ethical practices of everyday life – how we treat each other, and what kinds of people we are.

Hughes, a former UK medical consultant writing against the backdrop of dilemmas for health care professionals in 2020, commented: ‘Now more than ever, in the midst of the COVID-19 pandemic, we need the virtues and the insights that virtue ethics afford us’.^3^ He suggested medical professionals might ask whether they could be gentle and humane in their decisions, or brave in conveying bad news, for example. Two articles by theologians, writing in the field of public health, argue for the virtues of compassion^4^ and solidarity^5^ in response to Covid-19. However, while calling for and asserting the importance of virtues and virtue ethics, none of these articles offers a coherent account of virtues or virtue ethics. This gap is filled by Fowers et al. in a psychology journal article,^6^ which develops a carefully argued case for the role of virtues during Covid-19. They highlight the roles of courage, justice and practical wisdom in pandemic conditions of increased risk, injustice and complexity. Arguing from the perspective of neo-Aristotelian virtue ethics, they draw on some of the research and scholarship of the UK-based Jubilee Centre for Character and Virtues, in particular recent work on practical wisdom.^7,8^

## The approach of this article

The articles discussed above argue for the importance and usefulness of particular virtues and insights from virtue ethics based on generalised accounts of challenges faced in health care, public health and everyday life during the pandemic. In this article, I take a different and complementary approach, drawing on empirical research on specific, real-life ethical challenges reported by members of one profession – social workers. This enables understanding of the contextual details and dynamics of social professionals’ lived experience of risk, injustice and complexity, and how virtues might be called for and manifested in daily practice. It is important to stress that the empirical study, described in more detail later, was not designed as a study of virtues or virtue ethics per se. It was a study of the ethical challenges faced by social workers, based on self-reported written accounts in May 2020. However, it has relevance to the role of virtues during the pandemic as it contributes both a picture of the contexts in which social workers were practising (specific situations embodying high levels of risk, injustice and complexity) and their micro-ethical responses and reflections. Given the importance of context and particularity in virtue ethics, hopefully this paper can make a contribution to the literature on virtue ethics and the Covid-19 pandemic and will be of relevance to the health and caring professions more broadly.

## The role of social workers

While social workers have varied roles in different countries, usually they work with people needing social support or protection – including children, families, people with disabilities, community or neighbourhood groups. They often have statutory responsibilities to safeguard children and people with serious mental health and capacity issues, sometimes undertake community work, and may be employed by local government, charitable or private organisations. Traditionally their work focuses on face-to-face encounters. There is a stress on having in-depth dialogue with people using services to assess their needs and capacities, developing trusting relationships, and preserving confidentiality of personal and sensitive information. In many types of social work, it is important for practitioners to be able to assess people’s living conditions, family circumstances, support networks and overall environment.

Different countries experienced rises in Covid-19 infection rates at different times and imposed restrictions on their services and citizens in varying degrees. However, by May 2020, the impact of Covid-19 was being felt, or anticipated, in most countries, with many introducing restrictions on people’s movement and contact. For social workers, this often entailed offices being closed initially, with requirements to work from home and undertake virtual or telephone meetings and assessments. Personal protective equipment was in short supply. Many support services to which social workers might refer people were unavailable, such as foster placements for children or day centres for people with disabilities (for a picture of conditions for social workers around the world and their responses, see Truell and Compton^9^; for the UK, see Kong et al.^10^).

## A qualitative survey on ethical challenges during Covid-19

In May 2020, the Social Work Ethics Research Group (an international group of academics) in partnership with the International Federation of Social Workers (IFSW) and British Association of Social Workers (BASW) conducted a qualitative online survey of ethical challenges faced by social workers globally during Covid-19. The project was coordinated by the author of this article, funded via Durham University. Ethical approval was granted by Durham University Department of Sociology Research Ethics Committee for all aspects of the study. Respondents gave informed consent for their anonymised responses to be used in research and publications and identifying features were kept confidential within the research team. The survey was designed to be simple to complete, with the aim of identifying ethical challenges and preparing guidance for practising ethically during pandemic conditions. In addition to seeking demographic and employment-related information, the survey comprised two main questions:

1. Briefly describe some of the ethical challenges you are facing/have faced during the Covid-19 outbreak? (Ethical challenges are situations that give you cause for professional concern, or when it is difficult to decide what is the right action to take. This may be a situation facing you, or something you have come to hear about from others).
2. Please give more details of a particular situation you found ethically challenging. This might be 1 to 2 pages long and might cover:

(a) *The background* to the situation: your role and responsibilities, the organisational context, any relevant legal or cultural issues.
(b) *What happened* and who was involved: what you and others said and did.
(c) *If you made a decision*, what was the decision and what was the reasoning behind it? Did you consult with anyone else?
(d) *What was your emotional response* (e.g. any positive or negative feelings)?
(e) *What further reflections* do you have on this situation afterwards?

Methodologically, the research took a narrative ethics approach – that is, seeking participants’ own qualitative accounts of their experiences, framed within an ethical lens. Narrative ethics places value on the use of stories as a way of eliciting first-hand accounts of people’s experiences of situations, which also serve to define and develop their ethical identities.^11^

Invitations to complete the online survey were distributed via IFSW’s website, mailing lists of national social work associations and other networks. There were 607 responses from 54 countries (for a list of countries see Banks et al. 2020^12^). All responses were translated into English, enabling the principal investigator (author of this article) to undertake a preliminary generic thematic analysis^13^ to identify broad types of ethical challenge. Different team members then analysed the nuances of the data in different languages, discussing findings in regular group meetings and agreeing the final typology of ethical challenges.^12,14^

Following publication of the overall findings, I undertook a further detailed analysis, focussing on the answers to question 2 (asking for details of a particular situation), to categorise the broad ways social workers reported responding to particular challenging situations. Their answers comprised narrative accounts of problematic situations, usually featuring the teller as an ethical agent, sometimes including accounts of motivations, intentions, emotions and reflections. Some were brief, others were more detailed. In examining each narrative as a whole, I considered its overall tenor as an account of ethical agency, what messages I picked up as a reader engaging with the narrative and, specifically for this article, what virtues were manifested in the accounts. Since the respondents themselves were not asked to identify virtues, I interpreted their accounts through a virtue ethical lens, looking for evidence of actions and reflections that might be regarded as just, caring, courageous, etc. As noted in Banks (2018),^15^ unless specifically invited to identify their own virtues in action, most people, including professionals, do not naturally speak in terms of virtues or character. They are more likely to describe actions. Hence, viewing accounts through a virtue ethical lens requires interpretation by the reader/researcher and familiarity with virtue ethics. I drew on my earlier conceptual work on specific virtues relevant to health and social care.^2^ As with all qualitative analysis, the interpretation is subjective. But reproducing verbatim responses in later sections of this article allows readers themselves to question and elaborate upon my interpretations.

As a backdrop to this article, the already-published findings on social workers’ ethical challenges and their responses^12,14,16^ are summarised in Lists 1 and 2.

### List 1: Types of ethical challenge faced by social workers during Covid-19 (May 2020)

1. Creating and maintaining trusting, honest and empathic relationships via phone or Internet with due regard to privacy and confidentiality, or in person with protective equipment
2. Prioritising service user needs and demands, which were greater and different due to the pandemic, when resources were stretched/unavailable and full assessments were often not possible
3. Balancing service user rights, needs and risks against personal risk to social workers and others, in order to provide services as well as possible
4. Deciding whether to follow national and organisational policies, procedures or guidance (existing or new) or to use professional discretion in circumstances where the policies seemed inappropriate, confused or lacking
5. Acknowledging and handling emotions, fatigue and the need for self-care, when working in unsafe and stressful circumstances
6. Using the lessons learned from working during the pandemic to rethink social work in the future

### List 2: Typology of social workers’ responses to ethical challenges during Covid-19 (May 2020)

• *Ethical confusion –* not knowing what was the right action to take, or how to work out what was right.
• *Ethical distress* – feeling negative emotions derived from knowing what would be the right course of action, but being unable to carry it out due to institutional or other constraints.
• *Ethical creativity* – making extra effort to work out what would be right in new circumstances, and being flexible and imaginative in carrying it out.
• *Ethical learning* – reflecting on learning from working during the pandemic and implications for ethical practice in the future.

These broad typologies were drawn from analysis of the international data. In examining how the challenges and responses were manifested in practice, I will illustrate with extracts from accounts given by the 41 UK social workers who responded to the survey, who were working within similar legal, policy and political contexts. In different countries, policy and legal contexts vary, as do social workers’ specific roles and responsibilities. The UK is used as a case example, representative of the underlying trends experienced internationally during the pandemic, played out in a local context. The more detailed work on the UK data was undertaken in partnership with BASW, with a view to developing policy and practice guidance, and was included in the original ethical approval.

## Illustrating the challenges in the UK context: Issues of range, intensity and visibility

The headline types of challenge in List 1 are recognisable as those that would be experienced in ‘normal’ times by most health and social care professionals – building trusting relationships, prioritising scarce resources, and handling risks and emotions. However, the detail behind them, signified by references to digital working, personal protective equipment and unavailability of assessments, indicates the changed conditions for practice. The ethical challenges faced during the pandemic were magnified across several dimensions: range, intensity and visibility. I discuss each in turn, illustrating with examples from UK social workers.

In terms of *range*, everyday situations not usually regarded as ethically problematic suddenly generated dilemmas (e.g. deciding whether to undertake a home visit) and risks were identified where none existed before (e.g. the risk of social workers passing on the virus to service users and their own families). As an adoption social worker commented: ‘I am used to assessing risk in others, but now I am a risk and potentially at risk’. Not surprisingly, situations like this could be found overwhelming, leading to ethical confusion not only over what was the right course of action, but also over what criteria should be used to decide what was right.

The *intensity* of the experience of ethical challenges also grew, as greater and more urgent needs, new demands and inability to meet demands caused heightened emotions, sometimes resulting in ethical distress. For example, social workers who usually worked in hospitals and assessed people’s coping and care needs prior to discharge, were no longer hospital-based, were unable to assess before discharge and were required urgently to find places in care homes without discharged patients or residents in homes being tested for Covid-19. As a senior manager in adult social care commented: ‘I have lost sleep over the decision-making I am seeing around me and the distress this is causing frontline workers, my managers, families and carers’.

Regarding *visibility*, underlying ethical contradictions, inequities and injustices in society and social work practice were brought to the surface and exacerbated. People already experiencing poverty, discrimination or powerlessness often fared badly in terms of job loss, restrictions on movement or closure of services. Existing power imbalances, shortages of adequate resources and poor-quality services became more apparent. For example, some residents in care homes or other congregate settings were forbidden to leave home to shop or exercise, there was a shortage of foster placements and adequate accommodation for looked-after young people and some public services ceased. This meant social workers had to make great efforts to fill the gaps, respond to new and changing needs, challenge injustices, be more creative in finding solutions and be willing to accept inadequate or unjust solutions.

## Responding to the ethical challenges: The opening and narrowing of discretionary spaces

In responding to these challenging conditions, social workers had to recognise and act on both the expansion and contraction of spaces for professional discretion in decision-making and action. ‘Professional discretion’ can be understood as reasoning that results in judgements and actions in conditions of indeterminacy.^17,18^ Evans,^19,20^ writing in a social work context, distinguishes three types of discretion: *de jure* (power to act is officially sanctioned); de facto (having power to act, although not officially sanctioned); and entrepreneurial (acting outside policies and procedures, with managers informally allowing this). I am using the term ‘discretionary space’ to refer to the leeway for the use of professional discretion.

Pandemic conditions opened up spaces for professional discretion where none existed before, as offices closed, managerial guidance was limited and usual services and practices became unavailable. For example, a social worker in adult services, faced with a self-neglecting alcohol-dependant man referred by neighbours, decided to maintain welfare visits because the local authority environmental health team, which would usually deep clean such properties, was not operational. This was not in line with usual policy and practice, and therefore could be regarded as a case of de facto or entrepreneurial discretion, depending on whether the manager was aware and supportive.

At the same time, there was also a narrowing of other spaces, as blanket rules and restrictions were imposed where none had previously existed (e.g. restrictions on home visits and people’s freedom to move around). This is illustrated in the account given by another adult services social worker, who challenged the deputy manager of sheltered accommodation for not allowing a resident to do his own shopping. This had resulted in the man moving to a hotel. This social worker undertook careful advocacy on behalf of the man, invoking human rights and arranging a family group conference. The social worker was, in effect, calling on the manager and local authority to reinstate the space for *de jure* discretion (giving the manager the power to decide if exceptions might be made to the policy based on professional assessment of the circumstances of each case).

Practising ethically, therefore, required a capacity and willingness by social workers to use discretion in these new open spaces, and/or to call for use of discretion in the closed spaces of blanket rules.

## Virtues at work

In so far as being virtuous and acting virtuously is about cultivating and exercising dispositions to act well in particular contexts rather than simply following rules or procedures, Covid-19 conditions created spaces for virtues to be exercised and recognised. Crisis conditions created vacuums in rules and normal practices, and more isolated professionals had to make independent judgements and decisions without reference to managers, colleagues or rulebooks. The impact of Covid-19 also meant that formerly hidden and perhaps tacit processes of ethical evaluation and demeanour became explicit and visible. For example, normally a social worker working with a family to prepare them for adoption of a child and support them through the process cultivates a demeanour and does many small actions that embody fairness in assessment of parenting suitability and care in attending to prospective adoptive parents’ concerns and children’s needs. Only if difficulties emerge does the social worker become consciously aware of these ethical practices, as the worker may have to reflect on how to do things differently or account for or justify what they have done. They may have to reconsider what matters most in a situation, and whether doing what the law or agency rules require will contribute to human flourishing. This is exemplified by a UK adoption social worker’s account of the careful ethical decision-making and complex logistics involved in proceeding with a necessary and long-planned move of a baby from foster care to adoptive parents, in contravention of new ‘lock-down’ restrictions. As these examples suggest, the pandemic offers the chance to explore virtues in the micro-ethical practices of everyday professional life.

Everyday practice suddenly became problematic and required extra vigilance in seeing what the ethical issues were, rethinking what might be right in new circumstances and how this could be implemented, bearing in mind restrictions. The most obvious virtue required in such circumstances, and very evident in social workers’ accounts of their ethical challenges and responses, is practical wisdom or *phronesis*,^8^ often called ‘professional wisdom’ in the context of professional work (deliberating well about what to do). Professional wisdom is a meta-virtue, which plays a role in identifying salient ethical issues in a situation, integrating various considerations and virtues, entailing a process of reasoning and working out how to put ethical judgements into action.^15^ Fowers et al.^6^ also identified justice and courage as important for life during the pandemic generally. Certainly, these virtues were noticeable in social work as practitioners strived to respond fairly and rectify injustices exacerbated by the pandemic and acted courageously in making in-person visits and challenging or circumventing rules and restrictions. Further virtues for social work in ‘normal times’,^2^ which were also relevant during the pandemic, include care (noticing and responding to people’s needs and concerns); respectfulness (acknowledging people’s value); trustworthiness (behaving as relied upon) and integrity (holding to the values of the profession and balancing virtues).

Although I have argued that the pandemic created spaces for virtues and called for exercise of virtues to fill bureaucratic and managerial gaps, being virtuous during the pandemic was harder than usual. It required not just extra effort to notice and anticipate potential harms and infringements of rights and think through the right course of action, but also to implement any course of action. The easier response to some of the challenges faced would have been to do nothing or stick with existing inappropriate procedures or follow new rules and guidelines despite their inadequacy. In case example 1, none of these options was judged possible, so the social worker had to use the discretionary space created by the pandemic. This case is a verbatim account of a particular situation given by a male child protection social worker who responded to the survey.

### Case example 1: Deciding to meet children in the garden

I have a case in court where the children were at a critical phase of care planning. Their mother had made some significant progress and we were planning to return these children home to her care with extra support in place. Shortly before the final hearing the mother was involved in a serious police incident that meant it was clearly not safe enough to return the children to her care. The COVID-related issues here were that I had to weigh up telling these children that we were now scrapping the rehab[ilitation] plan by video call, which felt very impersonal and uncontaining, or potentially placing them at risk by visiting them. The guidance we have received from the Department for Education on home visits has also been extremely vague. It says we aren’t to conduct visits except in exceptional circumstances, but it doesn’t say what that is, so I had to rely on my professional judgement. I decided to visit these children and speak to them in the garden from a safe distance. This felt a bit strange but I was satisfied that it was the right thing to have done.

A related issue is that I would normally have tried to deliver news like this alongside the children’s mother as a united front. I am strongly committed to relationship-based practice and consider this kind of work crucial for helping children and families meet the reality of their situation in as positive and healing way as possible, so they have the best chance of preserving some kind of relationship in the future.

This account describes the impact of the pandemic on the social worker’s everyday practice. Although it would always be difficult to tell a mother and her children that they could not be reunited, pandemic restrictions on in-person contact added further complexity to the social worker’s decisions and actions. He had to ‘weigh up’ communicating with the children via an ‘impersonal’ video call against making a risky home visit. This is framed as a dilemma, which could not be resolved by referring to the Department for Education guidance, as this did not specify the ‘exceptional circumstances’ in which a home visit could be conducted. The social worker comments that he ‘had to rely on my professional judgement’. Although his account does not give details of his reasoning processes and emotions, using the words ‘had to’ suggests he may have expected more specific guidance. Although discretion is frequently used in social work about when and how to communicate with people, in child protection work there is also a lot of guidance and mandatory procedures designed to manage and minimise risk. Given the health risks of Covid-19, the social worker probably expected more guidance. Nevertheless, he made his own decision, arranging a garden meeting that would be in-person but minimised risk. He also concludes that he was satisfied that it was right, so clearly did not have any misgivings or regrets.

Although he does not dwell on this, care is implied in the consideration given to not being ‘impersonal and uncontaining’ (meaning he wanted to be personally present and supportive at this difficult time for the children). His reference in the last paragraph to his commitment to relationship-based practice suggests a concern for his integrity as a good social worker, trying to hold onto his values at a time when this was difficult.

While the social worker in case example 1 had to fill gaps in government guidance using his own judgement, the social worker in the next case example made great efforts to create space for discretion in the application of local authority restrictions on residents in children’s homes. Case example 2 is an extract from an account by a therapeutic social worker working with looked after children (LAC) in a local authority (the full account with a commentary can be found in Banks and von Köppen^21^).

### Case example 2: Challenging blanket rules in residential care - advocating for a looked-after young person

I work with a 15-year-old girl, Lisa, who is living in residential care. She has experienced significant domestic violence over a sustained period and has had a number of one-to-one sessions with me to help her manage the impact of this. Lisa has become conscious of the impact that experiencing domestic abuse has had upon her emotions and ability to regulate big feelings. One of Lisa’s strategies in managing anger was to go for a walk outside around the locality of the residential home. Following the lockdown in the UK, walking has been restricted to once per day. The young people in the residential home were advised that if they left unauthorised, they were likely to be arrested by local police. The policy of the local authority was to ensure that the young people firmly adhered to the ‘stay at home’ advice. Lisa complained that she needed to leave the home for a walk on the odd occasion that she felt anger rising. She cited the fact that staff in the home usually went with her or encouraged her to do so, and was therefore upset that this could not take place.

I agreed with Lisa’s position, that she needed to go for a walk outside when she felt herself getting angry and communicated with the team manager about Lisa’s concerns. The manager advised that the restriction on walking outside was the policy of the local authority management … He requested a written response with my thoughts about challenging this. Following a discussion with my colleagues/peer professionals, I submitted a response, which included the following:

Looked after Children (LAC) have all experienced developmental trauma to some degree and their emotional reaction and responses to any external stressful situation (such as this) are likely at times to be lacking the kind of understanding and reaction we would want. If we consider further that as LAC, they will have attachment difficulties; their own particular attachment strategy will be triggered when feeling stress or a sense that they are not safe in some way. This can be maladaptive, but as the young people are well known to staff, it is generally managed: e.g., needing to go for a walk around to cool off, if feeling angry.

The main issue at hand I felt was for local authority to adopt a more flexible and understanding response to particular young people …

This social worker clearly had a relationship of care with Lisa. He knew her, was attentive to her situation, concerned about her and felt responsible for her well-being. As a social worker, he respected the rights of all residents to be kept safe, but his role was to ensure that Lisa in particular remained safe and stable. Arguably the virtues of care and respectfulness were important in this case, alongside professional wisdom (judging how to approach the manager, working out what arguments to use). As with the previous case, extra effort had to be made to enable a usually easy everyday activity to take place.

## A threefold framework

These two cases give a flavour of the situations facing some social workers and how they responded. In reviewing all UK social workers’ accounts (and the international respondents), three features stood out, identified as the deployment of: ethical vigilance; ethical reasoning; and ethical logistics. These could be regarded as constitutive of the overarching virtue of professional wisdom,^15^ encapsulating the specific challenges of practising in pandemic (or other crisis) conditions. While other virtues were evident, particularly care, respectfulness and justice, professional wisdom is more discernible in the social workers’ accounts. This is probably because it underlies the process of moral evaluation and judgement and plays a role in coordinating the other virtues – adjudicating in cases of conflict.^8^

This framework was developed out of the survey research and subsequently presented with the aim of assisting social workers in thinking about ethical practice during the pandemic. It was published alongside case studies and discussion questions as part of a continuing professional development resource by BASW.^22^ The elements are described briefly below, along with notes about resonance with the two case examples in this article.

1. *Ethical vigilance* – being alert and sensitive to the ethical dimensions of practice, particularly when under pressure. This encapsulates ‘moral perception’ – the capacity to notice and foreground ethical issues that may be hidden, and to see situations from several perspectives. It also entails a heightened awareness of social workers’ own stress and exhaustion and the need to counteract the tendency to rush, make judgemental remarks or fail to see potential harms or infringements of rights.
*In the case examples*, both social workers were alert to possible harm in proceeding without fully thinking through the implications for the people for whom they had professional responsibility.
2.* Ethical reasoning* – deliberating about how to balance different needs, rights, responsibilities and risks; weighing harms and benefits; judging what is the right approach or course of action; and justifying any decisions made. Due to new risks and reduced services, more weight may be placed on public good, safety and minimising health risks than in ‘normal’ circumstances. Hence, the practice of slow, ethical reasoning is more important, as a process of rethinking and recalibration of values and principles has to happen.
*In the case examples,* processes of ethical reasoning were evident, with the child protection social worker working through possible options and the therapeutic social worker making an argument for Lisa’s needs and rights.
3.* Ethical logistics* – working strategically and practically to act on ethical judgements and decisions,promoting service users’ welfare and respecting their dignity and rights as far as possible in constrained circumstances. This often involves complex work-arounds and time-consuming processes, including making efforts to resist unfair or unnecessary restrictions and find creative solutions to resource shortages.
*In the case examples*, extra effort was made to ensure ethical decisions could be implemented – arranging a garden meeting in case 1, and working with the team manager and writing to the local authority in case 2.

## Concluding comments

This article shows how conditions created by Covid-19 demanded a rethinking of what counted as good decisions and right actions and how these could be achieved with reference to one of the caring professions (social work). This enabled aspects of the micro-ethics of everyday practice to be made visible to practitioners, observers and researchers. While the settings, roles and responsibilities of nurses and other health care workers differ from social work, the broad types of challenges they faced were similar. The relaxation or inapplicability of many normally mandated procedures and expected ways of proceeding meant many care professionals had to improvise, using professional judgement and discretion. This created space for the exercise of virtues, as opposed to following rules, and demonstrated the importance of virtues during crisis conditions, providing opportunities to learn from these experiences and reconsider recent trends in health and social care towards managerialism and the circumscribing of professional judgement and discretion.
